# Association of Vitamin D Metabolism Gene Polymorphisms with Autoimmunity: Evidence in Population Genetic Studies

**DOI:** 10.3390/ijms21249626

**Published:** 2020-12-17

**Authors:** Adolfo I. Ruiz-Ballesteros, Mónica R. Meza-Meza, Barbara Vizmanos-Lamotte, Isela Parra-Rojas, Ulises de la Cruz-Mosso

**Affiliations:** 1 Grupo de Inmunonutrición y Genómica Nutricional en las Enfermedades Autoinmunes, Centro Universitario de Ciencias de la Salud, Universidad de Guadalajara, Guadalajara Jalisco 44160, Mexico; adolfo.ruba@gmail.com (A.I.R.-B.); monimez28@hotmail.com (M.R.M.-M.); 2 Instituto de Investigación en Ciencias Biomédicas, Centro Universitario de Ciencias de la Salud, Universidad de Guadalajara, Guadalajara Jalisco 44340, Mexico; 3 Programa de Doctorado en Ciencias de la Nutrición Traslacional, Centro Universitario de Ciencias de la Salud, Universidad de Guadalajara, Guadalajara Jalisco 44340, Mexico; bvizmanos@yahoo.com.mx; 4 Programa de Doctorado en Ciencias Biomédicas Inmunología, Centro Universitario de Ciencias de la Salud, Universidad de Guadalajara, Guadalajara Jalisco 44340, Mexico; 5 Instituto de Nutrigenética y Nutrigenómica Traslacional, Centro Universitario de Ciencias de la Salud, Universidad de Guadalajara, Guadalajara Jalisco 44340, Mexico; 6 Laboratorio de Investigación en Obesidad y Diabetes, Facultad de Ciencias Químico-Biológicas, Universidad Autónoma de Guerrero, Chilpancingo de los Bravo Guerrero 39087, Mexico; iprojas@yahoo.com

**Keywords:** vitamin D polymorphisms, autoimmune disease, *GC*, *CYP2R1*, *CYP27B1*, VDR

## Abstract

A high prevalence of vitamin D (calcidiol) serum deficiency has been described in several autoimmune diseases, including multiple sclerosis (MS), rheumatoid arthritis (AR), and systemic lupus erythematosus (SLE). Vitamin D is a potent immunonutrient that through its main metabolite calcitriol, regulates the immunomodulation of macrophages, dendritic cells, T and B lymphocytes, which express the vitamin D receptor (VDR), and they produce and respond to calcitriol. Genetic association studies have shown that up to 65% of vitamin D serum variance may be explained due to genetic background. The 90% of genetic variability takes place in the form of single nucleotide polymorphisms (SNPs), and SNPs in genes related to vitamin D metabolism have been linked to influence the calcidiol serum levels, such as in the vitamin D binding protein (VDBP; rs2282679 *GC*), 25-hydroxylase (rs10751657 *CYP2R1*), 1α-hydroxylase (rs10877012, *CYP27B1*) and the vitamin D receptor (*FokI* (rs2228570), *BsmI* (rs1544410), *ApaI* (rs7975232), and *TaqI* (rs731236) *VDR)*. Therefore, the aim of this comprehensive literature review was to discuss the current findings of functional SNPs in *GC*, *CYP2R1*, *CYP27B1*, and *VDR* associated to genetic risk, and the most common clinical features of MS, RA, and SLE.

## 1. Introduction

The etiology and progression of autoimmune diseases (AIDs) are multifactorial and complex [1]. Genetic and environmental factors such as nutrients have been proposed to partially explain the pathophysiology progression of autoimmunity [2]. Notably, vitamin D regulates the growth and differentiation of various cells of the immune system such as macrophages, dendritic cells, T cells, and B cells, which are able to express the vitamin D receptor (VDR), produce and respond to the active form of vitamin D, calcitriol (1α,25(OH)_2_D_3_) [3].

In autoimmune diseases, epidemiological studies have reported a high prevalence of vitamin D deficiency by the quantification of calcidiol; this deficiency has been associated with worse disease clinical activity and progression of systemic lupus erythematosus (SLE), rheumatoid arthritis (RA), and multiple sclerosis (MS) diseases [4]. Likewise, several clinical trials and murine studies using cholecalciferol supplementation have described vitamin D′s immunomodulatory properties in autoimmune diseases [5]. Lower calcidiol serum levels are a strong predictor for worse MS clinical manifestations [6] as well as RA patients show lower calcidiol levels than control subjects (CS), and they have shown a negative association of calcidiol serum levels with clinical disease activity [7]. Regarding SLE patients, a higher prevalence of calcidiol serum deficiency than the general population has also been reported [8], and the lower serum calcidiol in SLE patients is associated with high clinical disease activity [9].

Calcidiol serum deficiency in patients with autoimmune diseases and the general population could be attributed to several factors, including lack of exposure to sunlight, skin pigmentation, sunscreen use, nutrient intake deficiencies, age, use of glucocorticoids, and the genetic background of the populations [10]. The potential roles of 35 genes that could modulate the vitamin D serum levels status have been reported in previous studies, highlighting that multiple single nucleotide polymorphisms (SNPs) in these genes are associated with lower calcidiol serum levels [11], such as the SNPs described in the vitamin D binding protein (VDBP; rs2282679 *GC*), 25-hydroxylase (rs10751657, *CYP2R1*), 1α-hydroxylase (rs10877012, *CYP27B1*) and the vitamin D receptor (*FokI* (rs2228570), *Bsml* (rs1544410), *Apal* (rs7975232), and *Taql* (rs731236) *VDR*). Therefore, the aim of this comprehensive literature review was to discuss the current findings of the functional SNPs in *GC*, *CYP2R1*, *CYP27B1*, and *VDR* related to genetic risk and the most common clinical features of MS, RA, and SLE.

## 2. Genetic Susceptibility to Autoimmune Diseases

Autoimmunity is characterized by an exacerbated immune response against self-antigen, causing autoimmune disease (AID) phenotype. Within the AIDs, 81 different immune disorders have been described and they affect around 4.5% of the human population [12], and the incidence and prevalence of AIDs have increased over the last 30 years [13].

Multiple factors including genetics, epigenetics, and environment have been associated with susceptibility to AIDs [14]. There is a high rate of AID concordance in monozygotic relatives and dizygotic twins or other family members [15]. In MS disease, monozygotic concordance is approximately 25–30%, with 3–5% rates for dizygotic pairs [16], and MS heritability of around 25–76% was reported [17], for RA, monozygotic twins’ concordance is around 15%, with 60% of heritability variance [18], and for in SLE, the concordance rates within monozygotic twins vary from 24% to 56%, and its heritability is around 66% [19], which highlights the role of genetic variability in susceptibility to AIDs.

The major histocompatibility complex (MHC) encoded by the human leukocyte antigen (HLA) locus in chromosome 6 was described with a central role in genetic susceptibility to AIDs. Molecules encoded in this region are essential in the adaptive immune response and antigens’ presentation for recognition by T cells [20]. It is worth noting that HLA genes display one of the highest allelic diversity compared to other protein families, which contributes to individual differences in immune response. In general, there are specific HLA allelic associations with each AID, and some HLA alleles predispose to common multiple AIDs [21]. HLA-DR and HLA-DQ were widely studied to establish the susceptibility or protective contribution to AIDs [22]. Among these, HLA-DRB1 was associated with genetic susceptibility and modulation of clinical manifestations severity of MS [23,24], RA [25], and SLE [26].

In RA, some HLA-DRB1 alleles encode a five amino acid sequence motif in residues 70–74 amino acids of the HLA-DRβ chain known as “shared epitope” and are associated with a worsening of RA clinical severity [27]. Besides, several HLA haplotypes have been described to participate in a bimodal way to susceptibility or protection for different AIDs. Such is the case of the HLA-DR15 (DRB1*15-DQB1*06-DQA1*01) haplotype, which was associated with MS and SLE susceptibility, but is a protective haplotype for type 1 diabetes [22]. Notably, vitamin D response elements (VDRE) were identified in the promoter region of the HLA-DRB1 gene, and its expression is modulated by calcitriol through VDR genomic signaling, strengthening the relationship between vitamin D and the pathophysiology of AIDs [28,29].

Apart from HLA genes, non-HLA genes have been proposed as susceptibility candidate genes for AIDs; these include genes related to modulating the immune response, such as cytotoxic T lymphocyte-associated antigen 4 (CTLA4), protein tyrosine phosphatase 22 (PTPN22), and tumor necrosis factor-alpha (TNF-α) [30].

SNPs reported in key enzymes and proteins involved in vitamin D metabolism such as rs2282679 in *GC*, rs10741657 in CYP2R1, rs10877012 in CYP27B1 and FokI (rs2228570), BsmI (rs1544410), ApaI (rs7975232), and TaqI (rs731236) in VDR, and the haplotypes aBF and ABF(or also interpreted as FBa and FBA according to their location in VDR gene), which means that this combination of alleles (a/A of ApaI, B of BsmI, F of FokI) are associated with genetic risk to SLE and higher clinical disease activity score, respectively [31]. However, it is important to mention that the relevance of the polymorphisms located in non-HLA genes lies in the functional effect that this exerts on the gene where it is located, which will be described in the following sections.

## 3. Genetic Polymorphisms Overview

Genetic polymorphisms are defined as heritable sequence alterations in the genome presented in more than 1% of the human population [32], which could modify genes’ expression or function. Therefore, they may affect biological pathways and susceptibility to a variety of diseases [14]. SNPs are present around every 1900 base pairs (bp) and appear relatively constant across the genome, except for the sex chromosomes [33]. SNPs are commonly classified as transitions in which a purine exchanges a purine (adenine “A” and guanine “G”) or a pyrimidine by pyrimidine (cytosine “C” and thymine “T”), or as transversions in which a pyrimidine exchanges a purine or conversely (A > C, A > T, G > C or G > T).

Transitional SNPs occurred 2.8-fold more often as transversions [34]. However, an SNP might be significantly associated with several different disorders, and its allele may contribute to susceptibility to one disease, but it may be protective for another [35]. Population genetic studies such as genomic wide association studies (GWAS), case-control, and cohort designs are essential to assess whether there is a potential association regarding the presence of SNPs variations in a specific healthy or sick population. However, only specific molecular genetic studies can reveal whether polymorphisms are functional or not [24,36] Therefore, based on molecular genetic studies, the functional effect of candidate SNPs was assessed recently, and even depending on the SNP location in their gene, several authors have hypothesized their putative function [37].

## 4. Functional Effects of Genetic Polymorphisms

Based on their location, several studies have described the different molecular functional effects of the SNPs depending on whether they are located in the promoter region, 5′ or 3′untranslated region (UTR), intron or exon region of the genes [38,39].

First, promoters are key gene regions involved in initiating transcription and act as *cis*-acting elements that can regulate gene expression. Polymorphisms found in promoter sequences are potential sources of gene expression rate modification, and a high proportion of promoter variants may modify gene expression by 50% or more [39]. SNPs in promoter binding regions may suppress gene expression, while others may only influence such genes’ rate expression [38].

**Figure 1:**
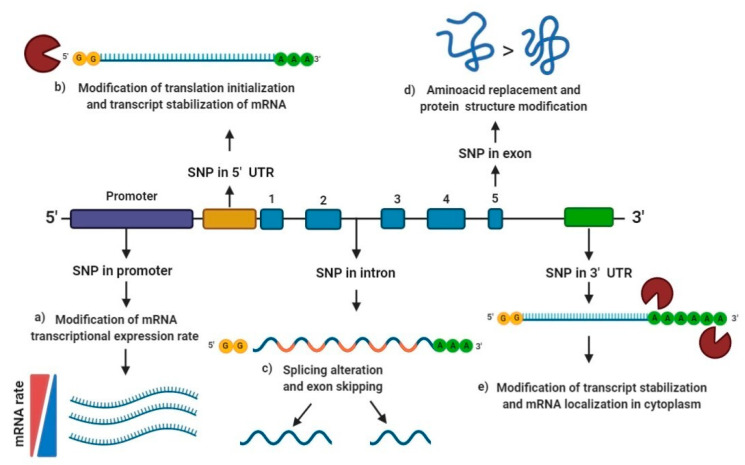
Functional effect of single nucleotide polymorphisms (SNPs) according to their location: (**a**) SNPs in promoter regions are reported to modulate gene expression by changing the conformation of the transcription factor binding site, may suppress gene expression, while others may only influence the expression of such gene; (**b**) SNPs in 5′ untranslated region (UTR) may modify translation initialization and transcript stabilization of messenger ribonucleic acid (mRNA); (**c**) SNPs in intron regions may generate splicing alteration, exon skipping, and modulate nuclear export, the rate of transcription and transcript stability; (**d**) SNPs in exon regions may cause the replacement of one amino acid for another, also known as non-synonymous polymorphism, which may generate a protein structure modification; (**e**) SNPs in 3′ UTR may modify transcript stabilization and mRNA localization in the cytoplasm. SNP: Single nucleotide polymorphism; UTR: Untranslated region; mRNA: messenger ribonucleic acid.

According to the Eukaryotic Promoter Database (EPD), around 29000 human promoter sequences have been described [40]. Most promoter SNPs are located close to the transcription start, binding sites frequency in gene promoter regions is 2–3 higher in the −50/−100 range than the −400/−2000 up-stream region [38]. Therefore, most transcription factor binding-sites are located within 250 bp of the initiation site. In addition, a higher frequency of “G > C” transversion substitution is observed due to the higher “*GC*” content in gene promoter regions (Figure 1a) [38].

During the transcription, introns are removed from the heterogeneous nuclear messenger ribonucleic acid (hnRNA) to form the mature messenger ribonucleic acid (mRNA), which is only formed by exons. In the human genome, introns are on average ten-fold longer than exons and thus constitute the majority of the genes [41]. Evidence suggests that SNPs introns could alter the transcription rate of genes by nuclear modulation export, transcript stability, and the nuclear translation [42]. When the splice-site is changed due to a mutation or polymorphism, the spliceosome usually goes to the next available legitimate splice-site (exon skipping) or selects the next best non-usual, splice-site in the proximity (cryptic splice-site utilization). Virtually all polymorphism in dinucleotide flanking consensus sequences (5′ GT and 3′ AG) for splicing cause either exon skipping or cryptic splice-site utilization, causing a severe reduction absence of normally spliced mRNA [43]. In addition, intron SNPs located around 30 bp far from the nearest splicing site are shown to modulate either the transcriptional activity or the splicing efficiency and alter the expression of alternative transcripts (Figure 1c) [44].

Regarding exon SNPs, they may cause the replacement of one amino acid for another, also known as non-synonymous polymorphisms, which may alter the conformation of the protein or enzyme itself (Figure 1d). On the other hand, synonymous polymorphisms in exons may cause splicing modifications. More than 76% of risk SNPs are within 3–69 bp of exon ends, and around 20%–45% of such SNPs affect splicing [37].

Moreover, the UTRs are located flanking either upstream (5′) close to the star codon AUG or downstream (3′) close to the end of coding region for transcription UAA, UAG, and UGA [45]. The average size of 5′ UTR regions is around 210 nucleotides (nts) with a range of 18–2803 nts, while for the 3′ UTR region, the average size is 1027 nts and a range of 21–8555 nts. UTR regions interact with proteins and other functional and regulatory compounds like ribosomes or microRNAs [46].

Around 12% of 5′ UTR and 36% of 3′ UTR vary between individuals [45]. 5′ UTR is involved in translation initialization and transcript stabilization (Figure 1b), while 3′ UTR takes part in the regulation of transcript stabilization and its localization in the cytoplasm (Figure 1e) [47], and Cis-acting mRNA elements located in 5’ UTRs and 3´UTRs act as post-transcriptional control of mRNA, translation efficiency, nucleo-cytoplasmic transport, subcellular localization and stability (Figure 1b,e) [48].

Moreover, when different polymorphisms are in the same chromosome and relatively close to each other, it is usually observed some degree of correlation or statistical association called linkage disequilibrium (LD), which is generated during the meiosis [24]. The LD phenomenon results in “haplotype blocks,” stretches of DNA defined by the presence of high LD among the SNPs present at the same chromosome. Two or more SNPs in LD in the same haplotype block can define haplotypes and specific combinations of allele variants across these SNPs. When alleles presented a strong LD, it indicates that both alleles of two different polymorphic sites in an SNP are segregated in blocks from one generation to another and may confer a similar risk to diseases [49].

Notably, some polymorphic alleles due to their proximity could present a strong linkage disequilibrium (LD) to other alleles at the same gene and may be segregated in a block of haplotypes. Individually, some SNPs could not provide a functional effect; however, if they are in allelic haplotype conformation with another risk allele that confers a functional effect, both could be associated with the same risk phenotype [50,51].

In order to evaluate the LD in SNPs, the most commonly measures used are the D′ and r^2^ and both have a range from 0 to 1, where 0 indicates no LD, and 1 complete LD, which means that the SNPs evaluated may be segregated together in a haplotype block [48,50]. Besides, haplotype structures may provide information regarding the human evolutionary history, identify genetic variants related to several human traits and conditions [51,52], and provide additional power to map genetic disease markers [53].

## 5. Vitamin D Status and Genetic Evidence in the Populations

Regarding vitamin D deficiency, several factors have been described, mainly the lack of exposure to sunlight, latitude, the season of the year, skin pigmentation, and use of sunscreen; other factors involved in vitamin D deficiency are diet, age, pharmacotherapy administered (antiepileptic and glucocorticoids), and particularly, several studies have described that genetic differences between individuals and populations such as genetic polymorphisms could influence the vitamin D deficiencies presented in all populations around the world [10].

Multiple SNPs in *GC*, *CYP2R1*, *CYP27B1*, and *VDR* genes are associated with lower calcidiol serum levels [11]. Moreover, vitamin D serum deficiencies are present in a high frequency in a healthy population, which could be related to the SNPs’ presence in these genes that may modify the response to supplementation of vitamin D in health and disease. A study conducted in healthy Iranian adolescents described the differential effect of the *CYP2R1* (rs10741657) A > G SNP on the supplementation of 50,000 UI of cholecalciferol weekly over 9 weeks, and showed that participants carrying the AA genotype presented 2.5-fold higher calcidiol serum levels in comparison to those that carrying the GG genotype (OR = 2.5 (1.4–4.4); *p =* 0.002) [54]. This is evidence of the role of polymorphisms in genes related to vitamin D metabolism in the variation of the response to vitamin D supplementation, even in healthy conditions.

## 6. Polymorphisms in the Main Key Genes Related to Vitamin D Metabolism

The skin produces around 80% of vitamin D in the form of cholecalciferol when exposed to ultraviolet B (UVB) light at a wavelength of 290–320 nm, and the remaining 20% is obtained from the diet as ergocalciferol from mushrooms or as cholecalciferol from fortified dairy products, fish, and eggs, mainly [55].

**Figure 2:**
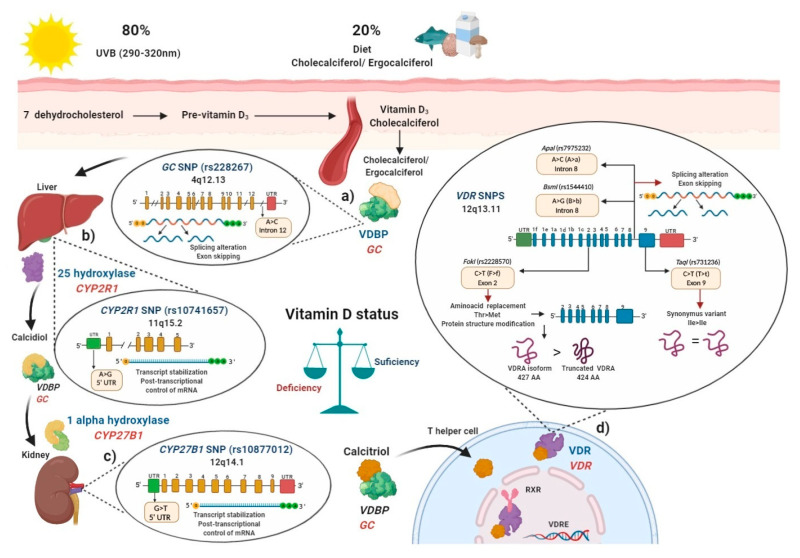
Polymorphisms in main key enzymes and proteins associated with vitamin D metabolism: localization and functional effects: (**a**) Vitamin D binding protein (VDBP) (encoded by *GC* gene) binds to ergocalciferol/cholecalciferol in order to be transported to the liver; *GC* (rs2282679) single nucleotide polymorphisms (SNP) due to its location in intron may generate a splicing alteration and exon skipping; (**b**) In the liver, 25 hydroxylase (encoded by *CYP2R1* gene) converts ergocalciferol and cholecalciferol to calcidiol and then calcidiol binds to VDBP to be transported to the kidney; *CYP2R1* (rs10741657)SNP located on the 5′ untranslated region (UTR) region may affect the transcript stabilization and the post-transcriptional control; (**c**) In the kidney, calcidiol is converted to calcitriol by the enzyme 1 alpha hydroxylase (encoded by the *CYP27B1* gene); *CYP27B1* (rs10877012) SNP located on 5′ UTR may affect the transcript stabilization and the post-transcriptional control of mRNA; (**d**) After calcitriol enters target cells and binds to vitamin D receptor (VDR) (encoded by *VDR* gene). Then, the VDR-calcitriol complex in the cytosol is translocated to the nucleus, where it binds to retinoid X receptor (RXR) to form a heterodimer, which interacts with vitamin D response element (VDRE) in vitamin D target genes, i.e., in T helper (Th) lymphocytes to suppress *IL-17A* or activate *FOXP3.* Mainly four SNPs have been described in the *VDR* gene: the *FokI* (rs2228570) located on exon 2, which generates a non-synonymous polymorphism with a change of C > T (also called F > f) and this results in a change of threonine to methionine. The presence of the restriction site *FokI* C allele (F allele), generates a new start codon (ATG) 9 bp after of the common starting site, which translate to an shorter truncated VDR protein of 424 amino acids with more transactivation capacity as a transcription factor than the wild type full-length VDR A isoform (VDRA) of 427 amino acids; the *BsmI* (rs1544410) located on intron 8 presents a change of A > G (also called B > b), could affect messenger ribonucleic acid (mRNA) stability and the gene expression of *VDR*, and also it could generate an alteration in the splice sites for mRNA transcription or a change in the intron regulatory elements of *VDR*; *ApaI* (rs7975232) located on intron 8 of *VDR* presents a change of A > C (also called A > a), does not change the amino acid sequence of the VDR protein, therefore could affect mRNA stability and the gene expression of *VDR*; *TaqI* (rs731236) is located on the exon 9 of *VDR*, presents a change of C > T (also called T > t) and generates a synonymous change of the isoleucine amino acid in the coding sequence, therefore it does not change the encoded protein, but it could influence the stability of the mRNA. All these SNPs are related to modulating de vitamin D serum status in health and disease. Ile: isoleucine; Thr: threonine; Met: methionine VDBP: vitamin D binding protein; VDR: vitamin D receptor; RXR: retinoid X receptor; VDRE: vitamin D response elements; UTR: untranslated region; THEM4: thioesterase superfamily member 4; Th: T helper lymphocyte; VDRA: wild type full-length VDR A isoform.

In blood, cholecalciferol and ergocalciferol bind to the vitamin D binding protein (VDBP), a protein encoded by the *GC* gene to be transported to the liver (Figure 2a). The *GC* (rs2282679) SNP was been associated with modulation of vitamin D serum levels in several populations [56,57,58]. In these studies, the main vitamin D circulating metabolite in blood, calcidiol, was used to evaluate vitamin D deficiencies [59].

In the liver, cholecalciferol and ergocalciferol are converted to calcidiol by the enzyme vitamin D 25-hydroxylase which is encoded by the *CYP2R1* gene (Figure 2b), the (rs10741657) SNP described in *CYP2R1* had also been related to modulate calcidiol serum status in several studies [54,56,57,60]. Then, after the generation of calcidiol in the liver, it binds again to VDBP and subsequently interacts with the enzyme 1-α hydroxylase, encoded by the *CYP27B1* gene, mainly in the proximal kidney tubule, calcidiol is converted to calcitriol (1α,25 dihydroxyvitamin D), which is the biological and functional active form of vitamin D (Figure 2c) [55]. Polymorphisms in *CYP27B1*, particularly the rs10877012 SNP, was associated with lower calcidiol serum levels [61].

Once the generation of calcitriol is performed, it is bound to VDBP and is transported to target cells and tissues where in cytoplasm or membrane, it interacts with the vitamin D receptor (VDR), encoded by the *VDR* gene.

After the calcitriol translocation to the nucleus, this complex forms a heterodimer with the retinoid x receptor (RXR), and thus regulates vitamin D target genes through its binding to specific DNA sequences called vitamin D response elements (VDREs) [62,63]. Since VDR is expressed in different immune cells, such as neutrophils, macrophages, dendritic cells (CDs), T and B lymphocytes, calcitriol may regulate the immune system [8].

Calcitriol can be produced by monocytes and macrophages and generate a shift from pro-inflammatory to tolerogenic immune status [64]. Calcitriol promotes M1 phenotype switching to M2 via the nVDR-PPARγ pathway and via the upregulation of the expression of IL-10 [65]. Particularly, calcitriol promotes a shift from Th1 and Th17 to Th2 immune profile via suppression of expression of cytokines of the Th1 (IL-2, IFN-γ, TNF-α) and Th17 (IL-17, IL-21) profiles, and induction of the expression of cytokines of Th2 profile (IL-4, IL-5, IL-9, IL-13), in order to limit inflammatory processes and autoimmune reactions [8,64].

Several studies have focused on evaluating SNPs described in the *VDR*, such as *FokI* (rs2228570), *BsmI* (rs1544410), *ApaI* (rs7975232), and *TaqI* (rs731236) polymorphisms, which was associated in several populations with lower calcidiol levels (Figure 2d). Therefore, SNPs′ presence in key enzymes related to vitamin D metabolism may modulate calcidiol levels and calcitriol function; they may also modify the disease activity in MS, RA, and SLE through the vitamin D deficiency, and contribute to genetic susceptibility to autoimmunity. In the following sections, we will describe how polymorphisms in these genes can modulate their expressions.

### 6.1. Vitamin D Binding Protein (VDBP) (SNP rs2282679 *GC*)

Vitamin D binding protein (VDBP) is the main carrier protein for vitamin D, which binds 85 to 90% of the total circulating calcidiol [66]. This protein is encoded by the *GC* gene located in chromosome 4, position 13.3 (4q13.3), and has 55,136 bp size [67]. The *GC* gene consists of 13 exons and the rs2282679 SNP was described located at the position 71,742, presenting an A ˃ C change (ancestral allele: A) in the intron 12, near to the actin III subdomain [68] and the endonuclease enzyme commonly used to identify this *GC* (rs2282679) SNP is *FokI* (*Flavobacterium okeanokoites*) [69]. According to rs2282679 SNP intron location, its hypothetical functional effect may alter genetic mRNA expression due to modification of the splicing process (44). A complete GWAS showed that *GC* (rs2282679) C allele was associated with lower serum calcidiol and VDBP levels in a study carried out in approximately 30,000 subjects with European ancestry included in 15 cohorts [70]. Additionally, various studies have shown the association of the *GC* (rs2282679) SNP with genetic susceptibility and disease modulation in MS, RA, and SLE [71,72,73,74] (Figure 2a).

### 6.2. Vitamin D 25-Hydroxylase (SNP rs10741657 CYP2R1)

The enzyme vitamin D 25-hydroxylase is a protein of around 500 amino acids with a molecular weight of 50–55 kilo-daltons (kDa). The liver is the main site where it is synthesized and performs its function of 25-hydroxylation enzyme activity, besides this enzyme activity was also described in the kidney and intestines [75], but cholecalciferol and ergocalciferol are metabolized mainly by the vitamin D 25-hydroxylase to calcidiol in the liver [76].

The enzyme vitamin D 25-hydroxylase is encoded by the *CYP2R1* gene located in chromosome 11 in the short arm at position 15.2 (11p15.2) and consists of 15,500 bp. The SNP rs10741657 *CYP2R1* is positioned at 14,893,332 pb in the gene, it displays an A ˃ G change in the 5′ UTR region (ancestral allele: A) [60].

In order to recognize the presence of this SNP, the endonuclease restriction enzyme *MnlI* (*Moraxella nonliquefaciens*) is commonly used [77]. According to the SNP location in 5′ UTR, the rs10741657 SNP may regulate gene expression by modifying translation initialization and transcript stabilization of mRNA, and therefore, modulate 25-hydroxylase expression and enzymatic activity rate [46].

A meta-analysis that included 16 articles with 52,417 participants showed that the *CYP2R1* (rs10741657) GG genotype shows trends of low calcidiol serum levels compared to the AA genotype in the Caucasian and Asian population [60], and when healthy persons are supplemented with cholecalciferol, carriers of the AA genotype showed a 2.5-fold increase in calcidiol levels compared to GG genotype carriers [54], which highlights the role of *CYP2R1* (rs10741657) SNP in vitamin D metabolism (Figure 2b).

### 6.3. Vitamin D 1-α Hydroxylase (SNP rs10877012 CYP27B1)

The enzyme vitamin D 1-α hydroxylase corresponds to a P450 protein of 507 amino acids of around 55 kDa [78]. This enzyme metabolizes calcidiol to calcitriol, the active form of vitamin D [76], and it is encoded by the *CYP27B1* gene, which is found in chromosome 12, long arm, position 14.1 (12q14.1), its size is 6653 bp.

The SNP rs10877012 *CYP27B1* is located at the non-coding region -1260 and is characterized by a change of G ˃ T (ancestral allele: G) [79,80]. The restriction endonuclease enzyme *HinfI* (*Haemophilus influenza)* is commonly used for its detection [69,77]. Due to its location in the 3′ UTR region, this SNP may alter transcript stabilization regulation and its localization in the cytoplasm [46].

A study carried out in 253 German patients with differentiated thyroid carcinoma showed that patients with the presence of the GG genotype (Referred as CC in this study by its position in the negative DNA strand) was associated with lower calcitriol serum levels than patients carrying the TT genotype (Referred as AA in this study by its position in negative DNA strand) (60 pmol/mL vs. 72 pmol/mL, respectively) [79].

In another study carried out in Caucasian German patients with gestational diabetes, the GG (CC) genotype was also associated with lower calcidiol levels [61]. Likewise, another study carried out in a healthy Caucasian British population demonstrated an association between the *CYP27B1* (rs10877012) G (C) allele and lower serum calcidiol levels [81].

Besides, in type 1 diabetes in Caucasian patients from Germany, those carrying the GG (CC) genotype had a reduced amount of mRNA from *CYP27B1* compared to HS (1.6855 vs. 1.8107, respectively, *p* = 0.0220) [61]. Therefore, the SNP rs10877012 *CYP27B1* may also modulate the vitamin D serum status and the genetic susceptibility or disease modulation in autoimmune diseases such as MS, RA, and SLE (Figure 2c).

### 6.4. Polymorphisms in Vitamin D Receptor (VDR)

Most genetic studies evaluating the potential association of calcidiol serum levels with genetic polymorphisms have focused on evaluating the polymorphisms described in the vitamin D receptor (VDR). The VDR is a member of the steroid/thyroid hormone receptor superfamily; and this receptor is encoded by the gene with the same name, *VDR*, which is located in chromosome 12, position 12q.13.11, comprising a region of approximately 100,000 bp of DNA, and only 4600 bp encode the VDR protein.

Functionally VDR is a transcription factor regulated by ligand binding and possibly by phosphorylation events [82]. It is a soluble 427 amino acid protein located mainly in the nucleus, cell cytoplasm, and cellular membrane, from where it translocates to the nucleus through the microtubule system after interaction with its ligand, calcitriol [83].

VDR is expressed in various organs involved in calcium metabolism, immune cells, and the nervous system (5). Three isoforms of the VDR were described.

The most common is the VDRA isoform of 427 amino acids and 48 kDa, with a start site in exon 2. The second is a long VDRB1 isoform of 477 amino acids and 54 kDa, this isoform presents 50 amino acids more in the N-terminal domain by an ATG start site in the exon 1d, described in the human kidney as well as in intestinal and renal epithelial cell lines [83,84].

The third is a shorter VDRA isoform of 424 amino acids and greater transactivation capacity as a transcription factor, caused by the SNP *FokI* in the exon 2 [83].

More than 14 different polymorphisms were described in human *VDR*, which could influence the modulation of the response to calcitriol by binding to VDR. The four SNPs most frequently studied are: *FokI* (rs2228570), *BsmI* (rs1544410), *ApaI* (rs7975232), and *TaqI* (rs731236) [82,85]. These were related to modulating the vitamin D status independently (in alleles and risk genotypes) as well as in haplotypes and haplogenotypes, both in original articles and meta-analyses (Figure 2d).

#### 6.4.1. FokI (rs2228570) VDR SNP

*FokI* (rs2228570) *VDR* SNP, also referred as the start codon polymorphism (SCP), was defined using the *FokI* (*Flavobacterium okeanokoites*) restriction enzyme in a restriction fragment length polymorphism test (RFLP) [86]. *FokI* (rs2228570) is located in exon 2 and is considered a non-synonymous polymorphism, because the change of C > T (ancestral allele T), also referred as F > f change, which generates a non-synonymous change of threonine to methionine and dictates two potential translation initiation sites [85].

The presence of the restriction site *FokI* is when the C allele is presented (also called F allele by the cut of the *FokI* restriction enzyme), this C allele generates a new start codon (ATG) 9 bp after the common starting site, which translates to a shorter VDRA protein of 424 amino acids instead of the wild type full-length VDRA isoform of 427 amino acids [86].

*FokI VDR* SNP (rs2228570) was found to be functional, and the short 424 amino acid VDRA isoform is somewhat more active than the long VDRA isoform of 427 amino acid, in terms of its transactivation capacity as a transcription factor [87,88]. In the absence of the restriction site *FokI*, the T allele (also called f allele), translation begins at the first original site at the exon 2, and the VDRA of 427 amino acids is expressed, which is 1.7-fold less active in its transactivation capacity, and presents less stability [89,90,91,92].

#### 6.4.2. BsmI (rs1544410) and ApaI (rs7975232) VDR SNPs

*BsmI* (rs1544410) and *ApaI* (rs7975232) *VDR* SNPs were defined using the *BsmI* (*Bacillus stearothermophilus*) and the *ApaI* (*Acetobacter pasteurianus)* restriction enzyme, respectively in a RFLP test.

*BsmI* (rs1544410) *VDR* SNP, located in the intron 8, presents a change of A > G (also called B > b), and the ancestral allele is the G allele [90]. Regarding its functional effect, it could generate an alteration in the splice sites for mRNA transcription or a change in the intron regulatory elements of *VDR*. *ApaI (rs7975232) VDR* SNP, also is located in the intron 8, presents a change of A > C, (also called A > a), and the ancestral allele is the C allele [63]. Both SNPs are located at the 3′ end of the *VDR* and do not change the amino acid sequence of the VDR protein. Therefore, they could affect mRNA stability and the gene expression of *VDR* by LD [63,90].

#### 6.4.3. TaqI (rs731236) VDR SNP

*TaqI* (rs731236) *VDR* SNP, located in the exon 9, was defined using the *TaqI* (*Thermus aquaticus*) restriction enzyme, and presents a change of C > T, (also called T > t), and the ancestral allele is the C allele. *TaqI* generates a synonym change of the coding sequence; therefore, it does not produce an amino acid change of the encoded protein, but it could influence the stability of the mRNA [90].

If *TaqI* (rs731236) *VDR* SNP is in high LD with *ApaI* (rs7975232) *VDR* SNP, its functional effect is the possible modification of one of the zinc fingers of the nuclear signaling heterodimer that binds to the VDREs located in the target genes [93].

Due to the closeness of these four *VDR* SNPs, they were studied to determine their LD. In various populations of SLE, RA, and MS patients, the *BsmI* (rs1544410), *ApaI* (rs7975232), and *TaqI* (rs731236) *VDR* SNPs were described with a strong LD [94,95], which infers that the alleles of these three polymorphisms could segregate into haplotypes from one generation of persons to another. In the case of *FokI* (rs2228570), it was described in a low LD with the other three *VDR* SNPs, which suggests that *FokI* does not segregate in blocks with others downstream *VDR* SNPs [94,95]. However, because the genetic recombination points vary between populations, *FokI* was also studied in haplotypes together with the *BsmI* (rs1544410), *ApaI* (rs7975232), and *TaqI* (rs731236) *VDR* SNPs in several populations [96,97,98].

## 7. Polymorphisms in Main Vitamin D Metabolism Genes Associated with Autoimmune Diseases

### 7.1. Multiple Sclerosis (MS)

MS is a chronic neuroinflammatory autoimmune disease that affects the brain and spinal cord, patients with MS often present motor deficiencies, fatigue, pain, and cognitive deficits in this condition [99]. MS patients have been described with lower calcidiol serum levels than CS, and even low calcidiol serum levels are a strong predictor for developing MS [6].

We identified one study regarding *GC* (rs2282679) SNP in MS patients, in which the T allele was associated with 4.5 nmol/L higher serum levels of calcidiol. In this same cross-sectional study, the *CYP2R1* (rs10741657) SNP was also evaluated and the AA and AG genotypes were associated with 6.9 nmol/L higher serum levels of calcidiol than G allele carriers [71]. One study regarding *CYP27B1* (rs10877012) SNP was identified, evidencing genetic protection to MS for the T allele carriers (OR = 0.88) in a Caucasian Swedish population [80] (Table S1).

Regarding *VDR* polymorphisms, eighteen studies that evaluated some or all *FokI* (rs2228570), *BsmI* (rs1544410)*, ApaI* (rs7975232), and *TaqI* (rs731236) *VDR* SNPs in MS were included in this review. Seventeen studies evaluating *FokI* (rs2228570) and MS were found, of which eleven studies did not find any significant association between this SNP and genetic susceptibility to MS, seven of them were case-control studies [100,101,102,103,104,105,106] and four of them were meta-analyses including overall, Asian, and Caucasian population [107,108,109,110] (Table S1).

In a case-control study in MS patients from the Netherlands, carriers of the *FokI* (rs2228570) FF genotype displayed lower calcidiol levels but also the F allele was associated with higher calcitriol levels [111]. Additionally, in a meta-analysis where thirteen case-control studies regarding *FokI* (rs2228570) were included, the FF and Ff genotypes (OR = 1.311) in a dominant genetic model, and the FF genotype (OR = 1.314) compared to the ff genotype were associated with genetic risk to MS in overall populations [112]. A case-control study evaluating only *FokI* (rs2228570) found an association of the Ff genotype (OR = 1.48) to MS in the Slovak population [113]. Conversely, a study found genetic susceptibility associated with the presence of *FokI* (rs2228570) ff genotype in a Portuguese population [114] (Table S1).

The majority of studies (eleven studies) regarding *FokI* (rs2228570) did not find any significant association to MS, either case-control studies [100,101,102,103,104,105,106] or meta-analyses [107,108,109,110]. However, no tendency for any specific genotype or allele was observed, one study showed that the FF genotype and F allele were associated with genetic risk to MS in the Caucasian European population [112], while a different meta-analysis displayed that the F allele, FF, and Ff genotypes were associated with protection to MS [115], while conversely, the ff genotype was associated to risk of MS in the Portuguese population [114] (Table S1).

Regarding *BsmI* (rs1544410) *VDR* SNP, ten studies were included in this review, of which six did not find any association between this SNP and genetic susceptibility to MS, three case-control studies [102,103,106], and three meta-analyses [109,110,112]. In a Czech population, it seems that the B allele provides genetic susceptibility to MS only in men [105], and in the Asian population, a meta-analysis showed that the bb genotype provides genetic risk to MS (OR = 1.78) [107] (Table S1).

In addition, in two meta-analyses, the *BsmI* (rs1544410) BB genotype was associated with genetic protection to MS (OR = 0.722) in a recessive genetic model, in a >40 years age overall from the Asian and Caucasian population [115], and also in the Iranian population [108], similar to another case-control study in Slovenians where the AA (or BB) genotype in a recessive genetic model provided an OR = 0.59 [116]. The majority of studies (six studies) regarding *BsmI* (rs1544410) did not find a significant association with MS, three case-control studies [102,103,106], and three meta-analyses [109,110,112]. However, we observed some tendency towards the bb genotype and b allele acting as a genetic risk factor to MS, while the BB genotype and B allele act as genetic protection to MS (Table S1).

Ten studies evaluating *ApaI* were included; five of them did not find any significant association between rs7975232 *ApaI* and genetic susceptibility to MS, these were one meta-analysis in Caucasian, Asian, and overall populations [109] and four case-control studies [102,103,116,117]. Conversely, three studies showed that the presence of the A allele and the AA genotype in *ApaI* (rs7975232) provide genetic susceptibility to MS, these were two meta-analysis in the Asian and Caucasian population [110,112] and one case-control study in the Czech Republic population [105] (Table S1).

However, in a different meta-analysis, an inverse association was shown of the *ApaI* (rs7975232) A allele and AA genotype with decreased risk of MS in the Iranian population [108]. Nevertheless, in the Asian population included in a meta-analysis, the *ApaI* (rs7975232) AA and aa genotypes were associated with a protective effect to MS (OR = 0.743) in a homozygous genetic model [115]. Therefore, almost half of the studies (five studies) did not find any significant association between *ApaI* (rs7975232) and MS [102,103,109,116,117], and we did not find any clear tendency for genetic risk or protective effect to an allele or genotype in the rest of the studies (Table S1), which presented a differential association for MS in different populations, where in some, it was associated with protection and in others with risk, which could partially be influenced by the racial component of each population evaluated.

Concerning the *TaqI* (rs731236) *VDR* SNP, in twelve studies, six original articles [101,102,103,104,116,117], and three meta-analyses in the Asian and Caucasian population [109,110,112] did not find any association regarding this SNP and genetic susceptibility to MS. While in the Iranian population, the *TaqI* (rs731236) TT genotype seems to provide genetic protection to MS (OR = 0.28) in a homozygote genetic model [108], as well as in a meta-analysis it was shown that in the Asian and Caucasian population, the TT and tt genotypes were associated with MS genetic protection in a homozygous genetic model [115], but in another meta-analysis, the Tt genotype was associated with MS risk compared to the TT genotype in overall populations [107] (Table S1).

The majority of studies (nine studies) regarding *TaqI* (rs731236) did not find any significant association to MS [101,102,103,104,109,110,112,116,117], and we did not observe a clear tendency on any allele or genotype to MS risk (Table S1). According to the data observed in several studies, we could conclude that in *TaqI* (rs731236), homozygous genotypes (TT and tt) were associated with protection, and the heterozygous genotype (Tt) was associated with risk to MS.

As previously mentioned, SNPs in the *VDR* gene may be segregated in haplotype blocks; therefore, we also include studies regarding haplotypes in *FokI* (rs2228570), *BsmI* (rs1544410), *ApaI* (rs7975232), and *TaqI* (rs731236) in MS patients. A Russian study that evaluated the *FokI* (rs2228570), *BsmI* (rs1544410), and *TaqI* (rs731236) *VDR* SNPs reported that the *TaqI* (rs731236) t allele and the Bft haplotype (also interpreted as fBt according to the order of location of the polymorphic sites in *VDR*) could increase the susceptibility to MS and may influence the clinical manifestations of MS [98].

In another study in Japanese MS patients and CS, where the *ApaI* (rs7975232) and *BsmI* (rs1544410) *VDR* SNPs and their association with *HLA* class II alleles were evaluated, the bA haplotype was more frequent in MS patients (OR = 10.39 [95% CI = 2.89–36.71], *p* = 0.0003) than in CS, and in bA carriers MS patients, the positive rate of DPB1*0501 was higher than in bA haplotype carriers controls subjects and bA haplotype non-carriers MS patients (*p* = 0.0308 and *p* = 0.0033, respectively) and the frequency of DRB1*1501 was higher in the [A] allele-positive patients than in the [A] allele-positive controls (*p* = 0.043). Therefore, with these findings, the authors suggest that VDR SNPs may be associated with susceptibility to MS, and HLA alleles may correlate with risk for MS together with these VDR SNPs [118]. In a case-control study in Spanish MS patients, DRB1*1501 with MS association seemed to be modulated by VDR genotype. Optimal modulation detection was reached in AA and Aa genotypes (A+) together with Apa I and TT and TC (T+) of TaqI SNPs. A certain degree of modulation of ORs was detected in both markers (A+ = 1.361 vs. A− = 0.974; T+ = 1.265 vs. T− = 0.874). However, no statistical significance of this VDR SNPs dependent modulation and DRB1 15:01-MS was observed [119].

Hence, we concluded that in MS, the T allele in *GC* (rs2282679) SNP and the A allele in *CYP2R1* (rs10741657) were associated with higher calcidiol serum levels [71], and the T allele in *CYP27B1* (rs10877012) SNP may provide genetic protection in the Caucasian Swedish population to MS [80]. In all *VDR* SNPs, more than fifty percent of studies did not find a significant association to MS, we only observed a slight tendency in *BsmI* (rs1544410) regarding the BB genotype towards genetic protection to MS, while the bb genotype acting as a genetic risk factor to MS, about *ApaI* was little evidence, and the *TaqI* homozygous genotypes (TT and tt) were associated with protection, and the heterozygous genotype (Tt) was associated with risk to MS (Table S1).

### 7.2. Rheumatoid Arthritis (RA)

RA is a chronic and inflammatory joint autoimmune disease characterized by autoantibodies such as rheumatoid factor (RF), which is an immunoglobulin (Ig) produced against the Fc portion of IgG; also, the presence of anti-cyclic citrullinated peptide antibodies (anti-CCP) is commonly observed in this condition. RA can lead to the accumulation of joint damage and irreversible disability [120]. RA patients have lower calcidiol serum levels than CS and a negative association in calcidiol serum levels with RA disease activity was described [7].

Three studies evaluating *GC* (rs2282679) SNP were found in this review. In the first study, a cross-sectional study, the *GC* (rs2282679) C allele was associated with lower calcidiol levels and the CC genotype (OR = 2.52) with hip fracture occurrence in Japanese RA patients [73]. The second study, a case-control study in northern China, found a significant association of the C allele with RA genetic risk (*p* = 0.026) [72]. Lastly, a GWAS meta-analysis study did not find a genetic association of *GC* (rs2282679) with RA occurrence in European ancestry populations [74] (Table S2).

Regarding *CYP2R1* (rs10741657) SNP, one study was found, in which the GG genotype was associated with lower calcidiol levels in RA patients from Spain [121] (Table S2). No studies regarding *CYP27B1* (rs10877012) SNP and RA were found in the literature.

Regarding the four *VDR* polymorphisms, seventeen studies evaluating any or all the four *VDR* polymorphisms were included in this review, nine evaluating *FokI* (rs2228570), eleven *BsmI* (rs1544410), four *ApaI* (rs7975232), and twelve *TaqI* (rs731236). Of the twelve studies evaluating *FokI* (rs2228570) *VDR* SNP and RA, two case-control studies in the Egyptian population found no significant evidence regarding genetic susceptibility [122,123].

The *FokI* (rs2228570) F allele and FF genotype were associated with genetic RA susceptibility in three case-control studies [124,125,126] and three meta-analyses [127,128,129]. In a case-control study in eastern Iran, *FokI* (rs2228570) Ff genotype (OR = 1.68) compared to FF genotype as well as Ff and ff genotypes (OR = 1.86) were associated with RA in a dominant genetic model [130]. In a cross-sectional study, the *FokI* (rs2228570) TT (or ff) genotype was associated with higher clinical disease activity in French and Tunisian RA patients [131] (Table S2). Therefore, the majority of studies (seven studies out of nine) found a significant association of *FokI* (rs2228570) and RA risk, and we observed a tendency where the F allele, FF, and Ff genotypes were associated with RA genetic risk in five studies [124,125,126,127,128,132], while the ff genotype seems to be associated with higher disease activity [131].

Concerning *BsmI* (rs1544410) *VDR* SNP, eleven studies were included, two cross-sectional studies [125,126] did not find any significant association regarding this SNP to RA risk, including one meta-analysis [127]. In the African population, the *BsmI* (rs1544410) bb and Bb genotypes and the b allele were associated with RA’s genetic susceptibility. However, no difference was found in overall populations in this meta-analysis [133] (Table S2).

In a different cross-sectional study, the GG (also known bb) genotype was associated with higher disease activity in French and Tunisian RA patients [131]. The *BsmI* (rs1544410) B allele (OR = 0.779) and the Bb genotype (OR = 0.719) were associated with genetic protection to RA in a meta-analysis that includes Asian, Caucasian, and European Caucasian populations [128]. We observed that in *BsmI* (rs1544410), four studies did not find a significant association with RA [125,126,127,134]. A tendency was observed where it seems that the bb genotype may be associated with genetic risk for RA in the African population [133] and higher disease activity in French and Tunisian RA patients [131]. In contrast, the B allele and Bb genotype may confer genetic protection to RA in Asian, Caucasian populations [128] (Table S2).

About the *ApaI* (rs7975232) *VDR* SNP, seven studies in RA were included in this review, three case-control studies did not find a significant association with RA [134,135,136] and one meta-analysis in the European and Asian population [129].

The *ApaI* (rs7975232) Aa genotype could provide genetic protection to RA (OR = 0.76) compared to the AA genotype in a meta-analysis of overall populations [133]. In a cross-sectional study in Egypt, the *ApaI* (rs7975232) aa genotype was in a higher frequency in RA patients (*p* = 0.0042) [122] (Table S2). The majority of studies (five studies) did not find an association between *ApaI* (rs7975232) and RA, while the Aa genotype was associated with genetic protection to RA in overall populations [133], while in another study, the aa genotype was in higher frequency in RA patients [122]. However, no clear tendency was observed in whether an allele or genotype in *ApaI* (rs7975232) is associated with RA.

Lastly, eleven *TaqI* (rs731236) and RA association studies were included in this review. Two case-control studies [126,135] and two meta-analyses in Asian and European populations [127,129] did not find any significant association of this SNP with RA.

Regarding the *TaqI* (rs731236) SNP, the TT genotype was associated with genetic susceptibility to RA in a meta-analysis in Asian and Caucasian populations [128] as well as with lower calcidiol levels in a cross-sectional study in Jordanian RA patients [137]. Moreover, the *TaqI* (rs731236) tt genotype was associated with genetic protection to RA (OR = 0.32) in a meta-analysis in the African population (OR = 0.32) and Arab population (OR = 0.43) compared to the TT genotype, as well as the tt and Tt genotypes (OR = 0.50) in a dominant genetic model in Africans [133] (Table S2). Four studies included in this review regarding *TaqI* (rs731236) and RA did not find any significant association [126,127,129,135]. We observed a tendency regarding the TT genotype and RA susceptibility in overall populations [128], and lower calcidiol levels in Jordanian RA patients [137].

Regarding haplotypes in *FokI* (rs2228570) with *BsmI* (rs1544410), *ApaI* (rs7975232), and *TaqI* (rs731236) in RA patients, a study that assessed the four *VDR* SNPs reported that the frequency of carotid plaques was significantly higher in RA patients who carried the GATG (also interpreted as FbAt according to the order of location of the polymorphic sites in *VDR*). This haplotype conferred a significantly higher risk of having carotid plaques (OR = 1.56 [IC, 1.09–2.42], *p* = 0.009) compared with the ACCG (also interpreted as FBaT), which was the most common in the RA patients evaluated [96].

We concluded that according to the studies included in this review, that the *GC* (rs2282679) C allele was associated with genetic risk for RA in the northern China population [72], lower calcidiol levels, and hip fracture occurrence in Japanese RA patients [73]. However, no significant association was found in populations with European Caucasian ancestry [74]. The *CYP2R1* (rs10541657) GG genotype was associated with lower calcidiol levels in RA patients from Spain [121], and we observed a tendency about the presence of *FokI* (rs2228570) FF and Ff genotypes, *BsmI* (rs1544410) bb genotype, *TaqI* (rs731236) TT genotype with RA genetic risk; however, no clear tendency was observed in *ApaI* (rs7975232) *VDR* SNPs and RA.

### 7.3. Systemic Lupus Erythematosus (SLE)

SLE is a chronic autoimmune disorder characterized by the involvement of multiple organ systems, loss of tolerance to self-antigens, and dysregulated interferon-alpha (IFN-α) responses. SLE’s pathogenesis is multifactorial, with an irreversible loss of immune tolerance that characterizes the disease could be attributed to the interaction between multiple genetic and environmental risk factors [138]. SLE patients have a higher prevalence of calcidiol deficiency compared to the general population [8]. In addition, an association between lower calcidiol levels with greater disease activity was reported [9].

One GWAS study showed no significant association between *GC* (rs2282679) SNP and SLE [74]. No studies regarding *CYP2R1* (rs10541657) and *CYP27B1* (rs10877012) SNPs were found.

Sixteen studies regarding *VDR* SNPs were considered, including any or all the four *VDR* polymorphisms: *FokI* (rs2228570), *BsmI* (rs1544410), *ApaI* (rs7975232), and *TaqI* (rs731236). Regarding only *FokI* (rs2228570) *VDR* SNP, fifteen studies and SLE were included. Three studies did not find a significant association between *FokI* (rs2228570) and SLE, two of them were case-control studies [139,140], and one of them was a meta-analysis in the Asian and European population [129]. The F allele and FF genotype in *FokI* (rs2228570) were associated with SLE susceptibility in two case-control studies in the Egyptian population [31,141] and three meta-analyses in the Arab, Asian, and overall populations [142,143,144]. Besides, the FF genotype was associated with higher disease activity evaluated by the SLEDAI score (Systemic Lupus Erythematosus Disease Activity Index) and higher disease damage by SLICC-ACR-DI score (Systemic Lupus International Collaborating Clinics/American College of Rheumatology) in the Egypt population [82,141]. Besides, the *FokI* (rs2228570) FF genotype was associated with lower calcidiol levels in Brazilian SLE patients [145] and susceptibility to lupus nephritis in Egypt SLE patients [141]. Therefore, we observed a tendency toward the presence of F allele, FF, and Ff genotypes with SLE genetic susceptibility, higher disease activity, and lower calcidiol levels in SLE patients (Table S3).

Regarding *BsmI* (rs1544410) *VDR* SNP, eleven studies were included, of which four case-control studies did not show any significant association of this SNP to SLE genetic risk [139,145,146,147]. The *BsmI* (rs1544410) B allele and BB genotype were associated with SLE susceptibility in four meta-analyses worldwide where Asian, Caucasian, and Latin American populations were included [129,142,143,148], and one cross-sectional study in the Egypt population [31]. In contrast, the *BsmI* AA genotype reported in a study in Polish SLE patients was associated with higher antinuclear antibodies (ANAs) titles [147]. One study in the Bulgarian population associated the *BsmI* (rs1544410) Bb and bb genotypes (OR = 2.7) and b allele (OR = 2.0) to genetic susceptibility in SLE [149]. Overall, we observed a tendency where the B allele, in homozygous BB and heterozygous Bb genotypes in *BsmI* (rs1544410), was associated with genetic risk to SLE (Table S3).

Concerning *ApaI* (rs7975232) *VDR* SNP, eight studies in SLE were included, of which two case-control studies [139,146] and five meta-analyses [129,142,143,144,148] did not find any significant association to SLE genetic risk. The *ApaI* (rs7975232) A allele was associated with SLE genetic susceptibility in the Iranian population in a tAf (or fAt) *VDR* haplotype [95], while the aa genotype was associated with genetic protection to SLE in overall populations (OR = 0.77) [148] (Table S3). The majority of studies (six studies out of eight) did not find any significant association with SLE, and heterogeneity of results was observed. Therefore, *ApaI* (rs7975232) may not be associated to genetic risk to SLE individually; however, in interaction with other polymorphic sites, it may be a risk SNP.

Regarding *TaqI* (rs731236) *VDR* SNP, nine studies were included, and seven studies did not find any significant association with SLE genetic risk, two of them were case-control studies [139,146], while five of them were meta-analysis [129,142,143,144,148]. The *TaqI* (rs731236) t allele and Tt genotype were associated with genetic susceptibility to SLE in the Indian population [90]. In Portuguese SLE patients carrying the *TaqI* (rs731236) TT (also known as tt) genotype, higher SLICC scores than patients with CC (also known as TT) and CT (also known as Tt) genotypes was observed [146] (Table S3). The overall evidence shows that *TaqI* (rs731236) displays no significant association to genetic risk to SLE. However, the t allele and the Tt genotype may provide genetic risk in the Indian population [90], while the Tt genotype was associated with higher damage disease in Portuguese patients [146].

In addition to risk alleles and genotypes in key enzymes of vitamin D metabolism to SLE, several studies have analyzed these *VDR* polymorphisms in their haplotype conformation. In a study of genetic association in children with lupus nephritis from Colombia, *BsmI* (rs1544410), *ApaI* (rs7975232), and *TaqI* (rs731236) *VDR* SNPs presented a high LD (D′ = 0.807), in which no association between the genetic variables with the endotype of lupus nephritis was demonstrated [94]. In a southeast Iranian population of SLE patients and control subjects, where only *TaqI* (rs731236) and *ApaI* (rs7975232) *VDR* SNPs were reported in DL (D′ = 0.42). Nonetheless, these SNPs were evaluated in haplotypes also considering to *FokI* (rs2228570), the tAf haplotype (also interpreted as fAt according to the order of location of the polymorphic sites in *VDR*) was associated with a higher risk to SLE (OR = 2.7 [95% CI, 1.1–6.8], *p* = 0.025), along with *FokI* (rs2228570) Ff genotype (OR = 1.8 [95% CI, 1.1–3.1], *p* = 0.02) and the *TaqI* (rs731236) Tt genotype (OR = 2.8 [95% CI, 1.6–5], *p* = 0.0002) [95].

In Egyptian SLE patients vs. CS, the *VDR* SNPs were evaluated in haplotypes conformed to three polymorphic sites considering *ApaI* (rs7975232), *BsmI* (rs1544410), and *FokI* (rs2228570), reported that the aBF and ABF haplotypes (also interpreted as FBa and FBA, respectively) were highly more frequent in SLE patients than CS and were associated with SLE risk (OR = 2.5 [95% CI, 1.62–3.91], *p* = 0.008 and OR = 6.5 [95% CI, 3.11–13.84], *p* = 0.001, respectively). Further, the ABF haplotype (or FBA) was associated with higher SLE activity (SLEDAI = ≥11, *p* < 0.001) and lower calcidiol serum levels (18.9 ± 12.4 nmol/L, *p* = 0.006) [31].

In another Egyptian study population of SLE and osteoarthritis patients vs. CS, the *FokI* (rs2228570) ff genotype and the fb haplotype of the *FokI* (rs2228570) and *BsmI* (rs1544410) *VDR* SNPs were associated with a higher SLE disease activity and were significantly in higher frequency in SLE patients than osteoarthritis patients and CS [97].

The finding in these studies shows that one GWAS study showed no significant association between *GC* (rs2282679) SNP and SLE [74]. No studies regarding *CYP2R1* (rs10541657) or *CYP27B1* (rs10877012) SNPs were found, and we observed that the F allele, FF, and Ff genotypes in *FokI* (rs2228570), as well as the B allele, BB, and Bb genotypes in *BsmI* (rs1544410), were associated with genetic risk to SLE. All studies showed no clear trend of association between *ApaI* (rs7975232) and *TaqI* (rs731236) to genetic risk for SLE.

## 8. Methods

### Literature Search Strategy

A comprehensive literature search was performed in the following databases and search engines: PubMed, Google Scholar, and Scielo. The most current and relevant information for each topic was included in this review. The following keywords were used to obtain information about the topics and subtopics: “autoimmune diseases” including; “systemic lupus erythematosus AND vitamin D”, “rheumatoid arthritis AND vitamin D”, “multiple sclerosis AND vitamin D”, “HLA AND/OR non-HLA susceptibility in autoimmune disease”, “Polymorphisms in Vitamin D metabolism genes” including; “*GC* rs2282679”, “*CYP2R1* 10741657”, “*CYP27B1* rs10877012”, “*VDR* polymorphism AND *FokI* rs2228570, *Bsml* rs1544410, *Apal* rs7975232, and *Taql* rs731236” and “*VDR* haplotypes”. In addition, all polymorphisms were searched paired with the autoimmune diseases (i.e., *GC* rs2282679 AND multiple sclerosis). Likewise, the methodology and the quality of the articles were carefully reviewed, as well as a complementary bibliography of each selected article in order to find more relevant information.

## 9. Conclusions

Vitamin D deficiency is commonly observed in healthy populations and patients with autoimmune diseases, and the response to vitamin D supplementation may be modulated by SNPs’ presence in key genes involved in its metabolism [5]. Notably, few studies (seven studies) evaluate the association of the *GC* (rs2282679), *CYP2R1* (rs10751657), and *CYP27B1* (rs10877012) SNPs with MS, RA, or SLE, compared to several studies (fifty-four studies) assessing the *FokI* (rs2228570), *BsmI* (rs1544410), *ApaI* (rs7975232), and *TaqI* (rs731236) *VDR* SNPs in these autoimmune conditions.

In this review, we observed more consistent tendencies of genetic risk to the carriers of *VDR* SNPs in MS, RA, and SLE diseases. Concerning *VDR* SNPs, the F allele, FF, and Ff genotypes of *FokI* (rs2228570) may provide a genetic risk to RA and SLE. The bb genotype of *BsmI* (rs1544410) was associated with MS and RA genetic risk, while the B allele, BB, and Bb genotypes were associated with SLE genetic risk. The *ApaI* (rs7975232) alleles or genotypes did not tend to show an association with MS, RA, or SLE susceptibility or disease activity, while, the TT genotype of the *TaqI* (rs731236) SNP was associated with genetic risk to RA, and probably protection to MS.

These heterogeneous findings may be partially explained due to the contribution of different genetic recombination processes and ancestry backgrounds of each population assessed, which could influence the genetic associations reported [147]. Regarding this, the results reported are limited to the populations where the SNPs were assessed. Therefore, the characterization of the population ancestry must be taken into account in each genetic study carried out, in order to validate the associations reported between the presence of SNPs in key genes involved in vitamin D metabolism with genetic susceptibility risk to autoimmune diseases.

## Supplementary Materials

The following are available online at https://www.mdpi.com/1422-0067/21/24/9626/s1. Table S1. Studies of polymorphisms in vitamin D metabolism genes and its association with multiple sclerosis; Table S2. Studies of polymorphisms in vitamin D metabolism genes and its association with rheumatoid arthritis; and Table S3. Studies of polymorphisms in vitamin D metabolism genes and its association with systemic lupus erythematosus.

## Abbreviations

AID	Autoimmune disease
CS	Control subject
HLA	Human leukocyte antigen
LD	Linkage disequilibrium
MS	Multiple sclerosis
OR	Odds ratio
RA	Rheumatoid arthritis
RNA	Ribonucleic acid
SLE	Systemic lupus erythematosus
SNP	Single nucleotide polymorphism
UTR	Untranslated region
VDBP	Vitamin D binding protein
VDR	Vitamin D receptor
