# Supplementary material for: Association of Vitamin D Metabolism Gene Polymorphisms with Autoimmunity: Evidence in Population Genetic Studies
Source: Int J Mol Sci. 2020 Dec 17;21(24):9626. doi: 10.3390/ijms21249626 (PMC7766382; doi:10.3390/ijms21249626)
Supplement: Supplementary file 1 [file ijms-21-09626-s001.pdf]

Table S1. Studies of polymorphisms in vitamin D metabolism genes and its association with multiple sclerosis.

Gene	Polymorphism	Population	Location and year	Study design	Association	Ref
GC	rs2282679	1497 MS patients	Denmark 2015	Cross-sectional	T allele was associated with 4.5 nmol/L higher calcidiol levels	(71)
CYP2R1	rs10741657				AA and AG genotypes were associated with 6.9 nmol/L higher calcidiol levels	(71)
CYP27B1	rs10877012	2158 MS patients and 1759 CS	Sweden 2010	Case-control	**Genetic risk to MS:** T allele (OR=0.88)	(80)
VDR	*Taq1* rs731236 *Fok1* rs2228570	727 MS patients and 604 CS	Australia and UK 2012	Case-control	**Genetic risk to MS:** *TaqI* t allele (OR =1.096)	(101)
	ApaI rs7975232 *TaqI* rs731236 *BsmI* rs1544410	270 MS patients and 303 CS	Slovenia 2016	Case-control	**Protection to MS:** *BsmI* AA (or BB genotype) (OR = 0.59) in a recessive genetic model	(117)
	Fok1 rs2228570	270 MS patients and 303 CS	Slovenia 2015	Case-control	**Genetic risk to MS:** *FokI* Ff genotype (OR =1.48)	(114)
	Fok1 rs2228570	533 MS patients and 446 CS	Portugal 2017	Case-control	**Genetic risk to MS:** *FokI* ff genotype (OR =1.687)	(115)
	Taq1 rs731236 *Fok1* rs2228570	303 MS patients and 310 CS	Spain 2013	Case-control	No genetic risk association to MS was found	(102)
	FokI rs2228570 *BsmI* rs1544410 *ApaI* rs7975232 *TaqI* rs731236	3300 MS patients and 3194 CS from 13 case– control studies	Asia, Australia, America 2014	Meta-analysis	**Genetic risk to MS:** *FokI* FF and Ff genotypes (OR = 1.311) in a dominant genetic model, and FF genotype (OR =1.314) compared to ff genotype in overall populations; *ApaI* AA genotype (OR = 1.468) compared to Aa genotype; AA genotype (OR=1.588) in a recessive genetic model; and AA and aa genotypes (OR=1.302) in a homozygous genetic model in overall populations	(113)
	FokI rs2228570 *BsmI* rs1544410 *ApaI* rs7975232 *TaqI* rs731236	1364 MS patients and 1661 unaffected first degree relatives	Canada 2011	Case-control	No genetic risk association to MS was found after Bonferroni correction	(103)
	FokI rs2228570 *BsmI* rs1544410 *ApaI* rs7975232 *TaqI* rs731236	30 case–control studies were included in the meta-analysis	Worldwide 2019	Meta-analysis	**Genetic risk to MS:** *BsmI* bb genotype (OR =1.78) in a recessive genetic model in Asian population; *TaqI* Tt genotype (OR = 1.27) compared to TT genotype in overall populations. **Protection to MS:** *ApaI* aa genotype (OR =0.61) in a recessive genetic model, and aa genotype (OR =0.52) compared to AA genotype in Asian Population	(108)
	FokI rs2228570 *BsmI* rs1544410 *ApaI* rs7975232 *TaqI* rs731236	214 MS patients and 428 age-matched CS	United states 2010	Case-control	No genetic risk association to MS was found	(104)
	FokI rs2228570 *ApaI* rs7975232 *TaqI* rs731236	167 MS patients and 146 CS	Turkey 2018	Case-control	**High frequency of risk genotype AA (FF) in MS patients *vs.* CS:** *FokI* AA (or FF) genotype: 9% *vs.* 4.1%, (p = 0.02); A (or F) allele: 32% *vs.* 19.9% (p = 0.001)	(118)
	FokI rs2228570 *BsmI* rs1544410 *ApaI* rs7975232 *TaqI* rs731236	80 MS patients and 50 age-matched CS	Iran 2015	Case-control	**Frequency differed significantly between MS patients *vs.* CS:** *BsmI* bb genotype: 21.25% *vs.*10% (p=0.023); *ApaI* Aa genotype: 66.25% *vs.* 44% (p=0.042)	(105)
	FokI rs2228570 *BsmI* rs1544410	113 MS patients and 122 CS	South East Iran 2015	Case-control	No genetic risk association to MS was found	(107)
	FokI rs2228570 *BsmI* rs1544410 *ApaI* rs7975232 *TaqI* rs731236	4013 MS patients and 4218 CS in 24 case-control studies meta-analyses	Asian and Caucasian 2018	Meta-analysis	**Genetic risk to MS:** *ApaI* A allele (OR = 1.267) in Asian populations	(111)
	FokI rs2228570 *BsmI* rs1544410 *ApaI* rs7975232	1206 MS patients and 1402 CS in 9 case-	Iranian population 2020	Meta-analysis	**Protection to MS:** *BsmI* B allele (OR= 0.69), BB genotype (OR=0.46) in a homozygote genetic model, and BB genotype (OR=0.56) in a recessive genetic model; *ApaI* allele A (OR =	(109)

TaqI rs731236	control studies were included			0.54) and AA genotype (OR = 0.28) in a homozygote genetic model, AA and Aa genotypes (OR = 0.56) in a dominant genetic model, and AA genotype (OR=0.35) in a recessive genetic model; *TaqI* TT genotype (OR = 0.28) in a homozygote genetic model	
FokI rs2228570<br>*BsmI* rs1544410<br>*ApaI* rs7975232<br>*TaqI* rs731236	296 Czech MS patients and 135 CS	Czech Republic 2018	Case-control	**Genetic risk to MS:** *BsmI* T (or B) allele (OR=2.05) in men; *TaqI* G (or t) allele (OR=1.9) in both; *ApaI* A (or A) allele in men (OR=2.05) and women (OR=1.42)<br>*BsmI* CC (bb) genotype (OR=3.02) was observed more frequent in women compared to men	(106)
FokI rs2228570<br>*BsmI* rs1544410<br>*ApaI* rs7975232<br>*TaqI* rs731236	21 relevant studies involving 3593 MS patients and 3917 CS meta-analysis	Caucasian and Asians 2017	Meta-analysis	**Protection to MS:** *FokI* F allele (OR=0.764), and FF and Ff genotypes (OR=0.417) in a dominant genetic model in publications after 2013 year; *BsmI* genotype BB (OR=0.722) in a recessive genetic model in the >40 years age group; *ApaI* AA and aa genotypes in Asian group (OR=0.743) and the 20.1-30°N latitude group (OR=0.609) in a homozygous genetic model; *TaqI* TT and tt genotypes (OR=0.846) in a homozygous genetic model, and TT genotype (OR=0.868) in a recessive genetic model in the 40.1-50°N latitude group	(116)
FokI rs2228570	212 MS patients and 289 CS	Netherlands 2009	Case-control	**Genetic risk to MS**: no association was found<br>**Lower calcidiol levels:** *FokI* FF genotype (*p*=0.024)<br>**Higher serum calcitriol in winter:** F allele (*p*= 0.034)	(112)
FokI rs2228570<br>*BsmI* rs1544410<br>*ApaI* rs7975232<br>*TaqI* rs731236	11 case–control studies involving a total of 2599 MS patients and 2816 CS	Worldwide 2012	Meta-analysis	No genetic risk association to MS was found	(110)
FokI rs2228570<br>*BsmI* rs1544410<br>*TaqI* rs731236	Russian MS patients	Russia 2009	Case control	**Haplotype Bft (or fBt):** Increase genetic risk to MS and clinical manifestations of MS	(99)
BsmI rs1544410<br>*ApaI* rs7975232	77 MS patients and 95 CS	Japan 2000	Case control	**Haplotype bA:** Genetic risk to MS**,** higher in MS patients than in controls (OR=10.39)	(119)

All odds ratios (OR) displayed on this table presented at least p <0.05. MS: multiple sclerosis; CS: control subjects; GC rs222679 (A>C); CYP2R1 rs10741657 (A>G); CYP27B1 (G>T) FokI rs2228570 (C>T / F>f) ; BsmI rs1544410 (A>G / B>b), ApaI rs7975232 (A>C / A>a), and TaqI rs731236 (C>T / T>t)

Table S2. Studies of polymorphisms in vitamin D metabolism genes and its association with rheumatoid arthritis.

Gene	Polymorphism	Population	Location and year	Study design	Association	Ref
GC	rs2282679	267 Chinese RA patients, 51 patients with AS (ankylosing spondylitis) and 160 CS	China 2012	Case-control	**Genetic risk to RA:** C allele (p=0.026)	(72)
		For RA, GWAS meta-analysis of 5539 autoantibody-positive RA patients and 20169 CS of European descent	Worldwide 2018	GWAS	No genetic risk association to RA was found	(74)
		1957 Japanese RA patients	Japan 2014	Cross-sectional	**Hip fracture:** CC genotype hazard ratio = 2.52 **Lower calcidiol levels:** minor C allele ($p = 8.1 \times 10^{-5}$)	(73)
CYP2R1	rs10741657	211 RA patients and 94 CS	Spain 2019	Case-control	**Lower calcidiol levels in RA patients:** GG genotype (19.70 ng/mL) compared to GA genotype (27.51 ng/mL) and AA genotype (26.16 ng/mL) (p =0.002)	(122)
CYP27B1	rs10877012	No studies were found				
VDR	*FokI* rs2228570 *BsmI* rs1544410 *TaqI* rs731236	1703 RA patients and 2635 CS in 12 case–control studies	Asian, Caucasian 2015	Meta-analysis	**Genetic risk for RA:** *FokI* FF genotype (OR = 1.762) compared to ff genotype; *TaqI* T allele (OR = 1.397), TT and tt genotypes (OR=1.643) in a homozygous genetic model, TT genotype (OR=1.899) compared to tt genotype, and TT genotype (OR=1.450) compared to Tt genotype in overall populations. **Probably protection to RA:** *BsmI* B allele (OR = 0.779), and Bb genotype (OR=0.719) compared to bb genotype in overall populations	(129)
	ApaI rs7975232 *TaqI* rs731236	151 Behcet`s disease patients, 106 AR patients and 179 CS	Tunisia 2014	Case-control	No genetic risk association to RA was found	(136)
	FokI rs2228570	448 native North American natives (NAN) RA patients and 704 NAN CS	Canada 2012	Case-control	**Genetic risk to RA:** *FokI* FF and Ff genotypes (OR=1.5) in a dominant genetic model	(125)
	FokI rs2228570 *BsmI* rs1544410	108 RA patients and 152 CS	Tunisia 2012	Case-control	**Genetic risk to RA:** *FokI* F allele (OR = 1.82)	(126)
	FokI rs2228570 *BsmI* rs1544410 *TaqI* rs731236	7 studies, 923 RA patients and 912 CS	Worldwide 2016	Meta-analysis	**Genetic risk to RA:** *FokI* F allele (OR =1.402) in Europeans	(128)
	FokI rs2228570 *BsmI* rs1544410 *TaqI* rs731236	100 RA French nuclear families and 100 additional French nuclear families for replication	France 2005	Case family based control design	**Higher frequency in RA patients:** *FokI* FF genotype compared to CS (45% *vs.* 30%, p=0.01)	(127)
	FokI rs2228570 *BsmI* rs1544410 *ApaI* rs7975232 *TaqI* rs731236	105 RA patients and 80 CS	Egypt 2015	Case-control	**Genetic risk to RA:** *BsmI* GG genotype (OR = 2.704) in a dominant genetic model; *ApaI* GG and GT genotypes (OR = 0.224) in a recessive genetic model; *TaqI* TT genotype (OR = 2.366) in a dominant genetic model	(124)
	TaqI rs731236	184 RA patients, 154 OA patients and 200 CS	Jordan 2018	Case-control	**Lower calcidiol levels:** *TaqI* TT genotype RA patients (11.67 ± 3. 24 ng/mL) compared to *TaqI* TT genotype CS (21.23 ± 3.43 ng/mL) (p =0.04)	(138)
	BsmI rs1544410	200 female RA patients and 150 CS	Egypt 2013	Case-control	**Lower bone mineral density:** *BsmI* BB genotype (p = 0.0001) in a recessive genetic model	(151)

BsmI rs1544410<br>*ApaI* rs7975232<br>*TaqI* rs731236	120 Spanish RA patients	Spain 2001	Cross-sectional	**Earlier onset form of rheumatoid arthritis:** *TaqI* tt genotype (28.80 ± 9.88 years) compared to Tt (44.29 ± 15.51 years) and TT genotypes (43.90 ± 11.75years) (*p*=0.04)	(135)
FokI rs2228570<br>*BsmI* rs1544410<br>*ApaI* rs7975232<br>*TaqI* rs731236	40 Italian RA patients	Italy Caucasian 2016	Cross-sectional	**Higher bone mineral density at the lumbar spine:** *TaqI* tt genotype (4.7%) compared to TT genotype (0.1%) (*p* <0.05)	(137)
FokI rs2228570<br>*BsmI* rs1544410<br>*ApaI* rs7975232<br>*TaqI* rs731236	40 RA osteoporosis patients, 88 RA no osteoporosis patients, 30 postmenopausal osteoporotic females and 150 CS from Egypt	Egypt 2014	Case-control	**Higher frequency in RA patients:** *ApaI* aa genotype (*p*=0.0042); *TaqI* TT genotype (*p*=<0.001).<br>**Genetic risk to RA:** *BmsI* b allele (OR=2.2); *TaqI* T allele (OR=2.26).<br>**Higher frequency in RA osteoporosis patients:** *FokI* Ff genotype (*p*=0.024) compared to RA no osteoporosis patients	(123)
FokI rs2228570<br>*BsmI* rs1544410<br>*ApaI* rs7975232<br>*TaqI* rs731236	Meta-analysis of 10 studies, 6 RA and 4 SLE studies	Europe and Asia 2011	Meta-analysis	**Genetic risk to RA:** *FokI* F allele (OR= 1.502)	(130)
FokI rs2228570<br>*TaqI* rs731236	30 RA patients and 128 CS	North East Iran<br><br>2019	Case-control	**Genetic risk to RA:** *FokI* Ff genotype (OR = 1.68) compared to FF genotype, Ff and ff genotypes (OR=1.86) in a dominant genetic model, and f allele (*p*=0.01); *TaqI* Tt and TT genotypes (OR = 1.79) in a dominant genetic model, T allele (*p*=0.01), and fT haplotype (OR=3.54)	(131)
FokI rs2228570<br>*BsmI* rs1544410	208 RA patients	France<br><br>and Tunisia 2014	Cross-sectional	**Higher disease activity DAS28:** *FokI* TT (or ff) genotype (*p*<0.001); *BsmI* GG (or bb) genotype (*p*<0.001)	(132)
FokI rs2228570<br>*BsmI* rs1544410<br>*ApaI* rs7975232	Meta-analysis included 23 eligible studies	Worldwide 2020	Meta-analysis	**Protection to RA:** *FokI* ff and Ff genotypes (OR=0.74) in a dominant genetic model, ff genotype (OR=0.66) compared to FF genotype, and Ff genotype (OR=0.85) compared to FF genotype in overall populations; *ApaI* Aa genotype (OR = 0.76) compared to AA genotype in overall populations; *TaqI* tt and Tt genotypes (OR=0.50) in a dominant genetic model, tt genotype (OR=0.44) in a recessive genetic model, tt genotype (OR=0.32) compared to TT genotype, Tt genotype (OR=0.57) compared to TT genotype, and t allele (OR=0.57) in Africans; *TaqI* tt genotype (OR=0.53) in a recessive genetic model, and tt genotype (OR=0.43) compared to TT genotype in Arabs<br>**Genetic risk to RA:** *BsmI* bb and Bb genotypes (OR= 1.82) in a dominant genetic model, bb genotype (OR=1.77) in a recessive genetic model, bb genotype (OR=2.40) compared to BB genotype, and b allele (OR =1.59) in Africans	(134)
FokI rs2228570<br>*BsmI* rs1544410<br>*ApaI* rs7975232<br>*TaqI* rs731236	591 Northern Spanish RA patients	Spain 2016	Cross-sectional	**Haplotype tAbF (or FbAt):** Higher risk to have carotid plaques (OR = 1.56)	(97)

All odds ratios (OR) displayed on this table presented at least p <0.05. RA: rheumatoid arthritis; CS: control subjects GC rs222679 (A>C); CYP2R1 rs10741657 (A>G); CYP27B1 (G>T)

FokI rs2228570 (C>T / F>f) ; BsmI rs1544410 (A>G / B>b), ApaI rs7975232 (A>C / A>a), and TaqI rs731236 (C>T / T>t).

Table S3. Studies of polymorphisms in vitamin D metabolism genes and its association with systemic lupus erythematosus.

Gene	Polymorphism	Population	Location and year	Study design	Association	Ref
GC	rs2282679	GWAS 1311 SLE patients and 1783 CS	European descent 2018	GWAS	No genetic risk association to SLE was found	(74)
VDR	*FokI* rs2228570<br>*BsmI* rs1544410<br>*ApaI* rs7975232	107 unrelated female SLE patients and 129 CS	Egypt 2013	Case-control	**Genetic risk to SLE:** *FokI* FF genotype (OR=4.9) and F allele (OR=1.9); *BsmI* Bb genotype (OR=2.5), BB genotype (OR=5.9) and B allele (OR=2.3); *ApaI* AA genotype (OR=2.8); aBF (or FBa) haplotype (OR=2.5) and ABF (or FBA) haplotype (OR=6.5).<br>**Higher SLEDAI scores:** *FokI* FF genotype and *BsmI* BB genotype (p<0.05); ABF (or FBA) haplotype (p<0.001)<br>**Lower serum calcidiol:** ABF (or FBA) haplotype (p=0.006)	(31)
	FokI rs2228570<br>*BsmI* rs1544410	54 SLE patients and 98 CS	Bulgaria 2016	Case-control	**Genetic risk to SLE:** *FokI* Ff and ff genotypes (OR=2.6) and f allele (OR=2.14); *BsmI* Bb and bb genotypes (OR= 2.7) and b allele (OR=2.0)<br>**Rash malar:** *BsmI* b allele (OR=2.5)	(150)
	FokI rs2228570<br>*BsmI* rs1544410	45 SLE patients and 40 CS	Egypt 2017	Case-control	**Lower calcidiol levels:** *FokI*: FF genotype compared to Ff and ff genotypes (p=0.001)<br>**Higher SLEDAI score:** *FokI* FF genotype compared to Ff and ff genotypes (p=0.02)<br>**Higher SLICC score:** *FokI* FF genotype compared to Ff and ff genotypes (p=0.002)	(82)
	FokI rs2228570<br>*TaqI* rs731236	331 female SLE patients and 282 CS	India 2018	Case-control	**Genetic risk to SLE:** *FokI* Ff genotype (OR=2.80), ff genotype (OR=2.57) and f allele (OR=1.96); *TaqI*: Tt genotype (OR=2.07) and t allele (OR=1.60)	(91)
	FokI rs2228570<br>*ApaI* rs7975232<br>*TaqI* rs731236	127 SLE patients and 139 CS	Southeast Iran 2019	Case-control	**Genetic risk to SLE:** *FokI*: Ff genotype (OR=1.80); *TaqI* Tt genotype (OR=2.80) and *FokI*, *ApaI* and *TaqI* tAf (or fAt) haplotype (OR=2.7)	(96)
	FokI rs2228570<br>*BsmI* rs1544410<br>*ApaI* rs7975232<br>*TaqI* rs731236	258 SLE patients and 545 CS	Poland 2013	Case-control	No genetic risk association to SLE was found.<br>**Renal disease:** *FokI* FF and Ff genotypes (OR = 3.228)	(140)
	FokI rs2228570<br>*BsmI* rs1544410<br>*ApaI* rs7975232<br>*TaqI* rs731236	11 studies with 1621 SLE patients and 1883 CS were included in this meta-analysis	Asia and Caucasian 2014	Meta-analysis	**Genetic risk to SLE:** *FokI* FF genotype (OR=1.469) compared to Ff and ff genotypes in Asians; *BsmI* B allele in overall populations (OR=1.726) and Asians (OR=1.952)	(143)
	FokI rs2228570<br>*ApaI* rs7975232<br>*TaqI* rs731236	12 studies meta-analysis, 1974 SLE patients and 2506 CS	Asia and Europe 2018	Meta-analysis	**Genetic risk to SLE:** *FokI* F allele in Arab population (OR = 1.721)	(145)
	FokI rs2228570<br>*BsmI* rs1544410<br>*ApaI* rs7975232<br>*TaqI* rs731236	Meta-analysis of 10 studies, 6 RA and 4 SLE studies	European and Asian 2011	Meta-analysis	**Genetic risk to SLE:** *BsmI* B allele in Asians (OR = 3.584)<br>**Lupus nephritis:** *BsmI* B allele (OR= 3.652) in Asians	(130)
	FokI rs2228570	52 SLE patients and 90 CS	China 2001	Case-control	No genetic risk association to SLE was found	(141)

FokI rs2228570<br>*BsmI* rs1544410<br>*ApaI* rs7975232<br>*TaqI* rs731236	13 studies in SLE patients and CS	Worldwide 2015	Meta-analysis	**Genetic risk to SLE:** *BsmI* B allele (OR=1.60) in overall populations<br>**Protection to SLE:** *FokI* ff genotype (OR=0.66) and f allele (OR=0.75); *BsmI* bb genotype (OR=0.51); *ApaI* aa genotype (OR=0.77) in overall populations	(149)
FokI rs2228570<br>*BsmI* rs1544410	195 SLE patients and 201 CS	Brazil 2012	Case-control	**Lower calcidiol levels in SLE:** *FokI* FF genotype compared to ff genotype (23.0 9.2 ng/ml *vs.* 31.6 ± 14.1 ng/ml; *p*=0.004)	(146)
FokI rs2228570<br>*BsmI* rs1544410<br>*ApaI* rs7975232<br>*TaqI* rs731236	170 SLE patients and 192 ethnicity paired CS	Portugal 2015	Case-control	**Higher SLICC:** *FokI* CT (or Ff) genotype compared to CC (or FF) and TT (or ff) genotypes (*p*= 0.031); *TaqI*: TT (or tt) genotype compared to CC (or TT) and CT (or Tt) genotypes (*p*=0.046)<br>No genetic risk association to SLE was found	(147)
FokI rs2228570	300 SLE patients and 300 age, sex, and ethnicity-matched CS	Egypt 2017	Case-control	**Genetic risk to SLE:** *FokI* F allele (OR = 1.6), and FF genotype (OR=2.7) compared to ff genotype<br>**Lupus nephritis:** *FokI* FF genotype (OR=4.8) compared to Ff and ff genotypes<br>**Higher SLEDAI-2K score:** *FokI* FF genotype compared to Ff and ff genotypes (*p*=0.01)<br>**Lower calcidiol levels:** *FokI* FF genotype compared to Ff and ff genotypes (*p*<0.01)	(142)
FokI rs2228570<br>*BsmI* rs1544410<br>*ApaI* rs7975232<br>*TaqI* rs731236	11 case–control studies of 1683 SLE patients and 1883 unrelated CS meta-analysis	Asian, Latin america and Europe 2014	Meta-analysis	**Genetic risk to SLE:** *FokI* FF and Ff genotypes in overall populations (OR=1.75) and Asians (OR=3.36) in a dominant genetic model; *BsmI* BB and Bb genotypes in overall populations (OR=2.14) and Asians (OR=2.86) in a dominant genetic model	(144)
BsmI rs1544410	62 SLE patients and 100 CS	Poland 2013	Case-control	**Higher levels of antinuclear antibodies (ANAs) in SLE:** *BsmI* AA genotype (*r* =0.438; *p* = 0.002) compared to GG and GA genotypes. No genetic risk association to SLE was found	(148)
FokI rs2228570<br>*BsmI* rs1544410	100 SLE patients, 100 osteoarthritic patients and 100 CS	Egypt 2017	Case-control	**Haplotype fb:** Higher frequency in SLE patients than CS (*p*=0.01)	(98)

All odds ratios (OR) displayed on this table presented at least p <0.05. SLE: systemic lupus erythematosus; CS: control subjects; GC rs222679 (A>C); CYP2R1 rs10741657 (A>G); CYP27B1 (G>T) FokI rs2228570 (C>T / F>f) ; BsmI rs1544410 (A>G / B>b), ApaI rs7975232 (A>C / A>a), and TaqI rs731236 (C>T / T>t).
